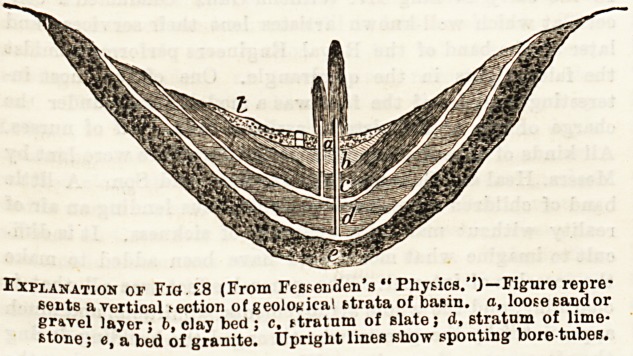# "The Hospital" Nursing Mirror

**Published:** 1896-07-11

**Authors:** 


"The Hospital" Nursing Mirror.

Being the Extra Nursing Supplement of "The Hospital" Newspaper.

[Contributions for this Supplement should be addressed to the Editor, The Hospital, 428, Strand, London, W.C., and should have the word "Nursing" plainly written in left-hand top corner of the envelope.]

### News from the Nursing World.

### THE ROYAL WEDDING.

We are glad to be able to announce that the marriage of the Princess Maud is now fixed for the 22nd inst., and is to take place in the Chapel Royal of Buckingham Palace. Our readers will be interested to hear that we intend reproducing a charming portrait of Princess Maud and Prince Charles on the 25th inst., together with an account of the wedding ceremony.

### KEPT AWAY BY DUTY.

"Never in my whole life have I had such a disappointment," owned a Queen's Nurse, who was obliged to relinquish at the last moment her long-looked-for visit to Windsor. The loss of an opportunity of seeing Her Majesty in her own beautiful home, and of going there in company with so many old friends and fellow workers, made up a disappointment hardly to be realised by those outside the nursing world. Yet there is a pleasant side to the picture, for we learn that it was her own strong sense of duty which kept the loyal little Cornishwoman at her post; the sick fisher folk needed her, and she knew it. So "cheerfully and most willingly, notwithstanding her disappointment, she went on with her work," writes one who knows her. The patients could not bear to think of her losing her treat, and besides "they wanted to hear all about the Queen from their own nurse"; and the doctors were equally sympathetic. In fact, one of the most striking features of this gathering together of the Queen's Nurses appears to be the kindly help which everybody gave, doctors, committees, and patients all striving to facilitate arrangements which would make things easy to each individual nurse.

### THE NURSES' CO-OPERATION "AT HOME."

A very successful conversazione was held by the committee of The Nurses' Co-operation (8, New Cavendish Street) at the Queen's Hall on Friday, July 3rd. Over eleven hundred guests put in an appearance during the afternoon, and were welcomed by Mr. J. P. Bickersteth, chairman of the committee, and Miss K. Philippa Hicks, lady superintendent. Amongst them were the Earl and Countess of Strafford, Sir William Fraser, Dr. Ray Lankester, the Hon. Mrs. Hugh Gough, Mr. Butlin, Mr. and Mrs. Burdett, Miss Gordon and Miss H. A. C. Gordon, Miss Davidson, Miss Squier, Miss de Pledge, and many other London matrons. Miss Rosalind Paget, Miss Gethen, Miss Brierly, Miss Dunn, Miss Oldham, and Miss Wade. The Meister Orchestra, under the direction of Mr. Eugene Maney, played excellently and gave much pleasure. This is the first gathering of the kind in connection with the Co-operation, and was a happy thought, which will help to keep up the pleasant spirit which now exists between the members of the Co-operation (one of the most successful of modern institutions), the public, and medical men. The nurses were each allowed to invite friends, and many of them were present themselves, adding much in the way of picturesqueness to the scene in their varied uniforms and spotless caps and aprons.

### MILDMAY DEACONESSES' HOME.

It is some forty years now since the Mildmay Deaconesses, now so well known, were first started in work at Barnet by the Rev. W. and Mrs. Pennfather. Many of these pioneer workers are now aged, and it is rightly desired that they shall be maintained in the evening of their days in the Memorial Home without being a burden on the already over-taxed funds of the mission. This Home is set aside for the use of all Mildmay workers in sickness and old age. A sale is to be held in aid of this good object, by kind permission of Mr. and Mrs. Elliot, in their garden at Hadley House, Barnet, on Thursday, July 16th, which it is earnestly hoped may be well attended. The sale will be open from three till eight o'clock. Frequent trains run from King's Cross to High Barnet and Hadley Wood stations.

### A WEDDING RING FROM WALES FOR PRINCESS MAUD.

It was proposed at the late annual meeting of the Eisteddfod Association, held at Llandudno, that the Association and the Gorsedd should present Princess Maud of Wales with a wedding ring made of Welsh gold. The suggestion, made by Sir John Puleston, was seconded by Lord Mostyn, and carried enthusiastically. Princess Maud will doubtless value her Welsh ring, and the loyal feeling which prompted so appropriate an idea.

### THE JUNIUS S. MORGAN BENEVOLENT FUND.

The honorary secretary of the above Fund asks us to state that several nurses have responded to the suggestion of Policy-holder of the Pension Fund No. 1,976 that they should send small subscriptions. If every member of the Pension Fund were to subscribe one shilling yearly the proceeds would support no fewer than seven necessitous nurses in their old age or when incapacited by illness. The honorary secretary asks us also to mention the kindly thought of a nurse retiring from the Fund upon her marriage, who has sent £1 1s. to the Fund.

### LIVERPOOL LADIES' CHARITY AND LYING-IN HOSPITAL.

At a meeting held on June 24th it was resolved that the medical officer on duty at the hospital should have sole and entire medical charge of all patients in the hospital, and that the medical board should be represented on the board of management by three members of the staff, who should have a right to vote.

### A CREDIT TO THEIR PROFESSION.

"Where are the nurses from Ireland?" asked someone the other day, scanning the ranks of uniformed figures gathered on Paddington platform, all eager anticipation at their visit to the Queen at Windsor.

"They are among quite the nicest looking of any here you will find," was the reply; and Miss Dunn and the two superintendents from the Dublin Homes (St. Patrick's and St. Lawrence's), Miss Howell and Miss St. Clair, might certainly have been congratulated on the group of bright-faced, neat looking nurses they brought over with them from "Ould Ireland." Many complimentary remarks might have been overheard on the appearance of all the nurses, who, in spite of the fact that some of them had come long distances already that morning, looked one and all fresh, and neat, and nurse-like. "Have they all come from the seaside?" said one spectator, "I never saw such a collection of healthy looking women before. District nursing is evidently good for the complexion!"

### GARDEN PARTY AT THE CANCER HOSPITAL.

Hospital authorities in London have been exceptionally active this season in the organisation of fêtes and entertainments in support of their respective institutions. The Chairman (Sir George S. Meason, J.P.) and Committee of the Cancer (Free) Hospital, Fulham Road, issued invitations to a garden party in the hospital grounds on Friday, June 26th, which was well attended. The band of the Royal Horse Guards (Blue) played during the afternoon, by permission of Colonel Brocklehurst.

### DISTRICT NURSES' HOME AT LEAMINGTON.

The Leamington District Nurses' Association have decided to take a house for their nurses, instead of providing them with furnished lodgings as hitherto. The committee have at present only sufficient furniture for one room, and much hope that their friends will be moved to help them by donations in money or kind. Suitable furniture would be gladly accepted.

### PLAISTOW DISTRICT NURSES' HOME.

The third annual meeting of the Plaistow Maternity Charity and District Nurses' Home was held, by permission of the Lord Mayor, at the Mansion House on Friday, July 3rd. The Earl of Winchilsea and Nottingham presided. The urgent need for adequate accommodation for the nurses was put before the audience, and a resolution was passed, moved by Lord Selborne, "That, in order to increase the efficiency and secure the economical working of the charity, it is of the utmost importance immediately to establish a building fund."

### TROUBLES AT BROMSGROVE.

The head nurse at Bromsgrove Union Infirmary has been requested to resign, in consequence of complaints made to the Board of alleged "unkindness" on her part to the inmates. From the remarks made before the Board by Dr. Kidd, the medical superintendent, it is evident that previous to Nurse Bumstead's appointment the infirmary was in a sad condition of neglect and discipline non-existent. He thought the present dissatisfaction was not from any fault on the nurse's part, but because she carried out her duties in a stricter way than the patients were accustomed to. He considered she had greatly improved the condition and management of the infirmary. It would appear that the complaints in question were through some misunderstanding of the doctor's orders, giving rise to the idea that Nurse Bumstead was "punishing" one of the patients by keeping her in bed. Nurse Bumstead has requested the Local Government Board to allow her a full inquiry into the facts of the case.

### NUNS AS NURSES.

In a letter to the *Guardian* dated June 24th, Miss C. J. Wood draws attention to the fact that in the Irish workhouses, as in foreign hospitals where the nursing is undertaken by Roman Catholic nuns, the value of their devoted care of the sick is sadly impaired for want of that systematic training which, according to the rules of their orders, they are not permitted to receive from the medical staff. These rules were doubtless drawn up before trained nurses existed, and an alteration would place at the disposal of the sick and infirm some of the most valuable material possible to obtain. The religious feeling and enthusiasm which leads these good women now to strain every nerve in the effort to alleviate suffering would render them most valuable nurses were they enabled to profit by modern knowledge, and undergo a complete training. At present experience is their only qualification.

### DIFFICULTIES AT LONGFORD.

The dispute between the Longford Board of Guardians and the Local Government Board over the question of nursing in the infirmary has at last come to an end, the sisters of mercy having settled the matter by undertaking the night duty. It is much to be regretted that the Local Government Board have not adhered to their original order for the appointment of a trained night nurse.

### AWARD OF MEDAL AND CLASP.

The Northern Workhouse Nursing Association have presented Nurse A. S. Raynor, of the Nottingham Union Infirmary, with a silver medal and clasp. This distinction is awarded by the association, according to custom, by permission of the guardians, to any nurse who has specially distinguished herself by devotion to her work, or who has remained in the employment of one board of guardians for three years.

### SHORT ITEMS.

The Duke and Duchess of York have fixed Saturday, July 25th, as the date of their visit to Halifax for the opening of the new infirmary, just completed, at Heath at a cost of £80,000.—Nine women candidates from Queen Margaret's College, Glasgow, entered for the final examinations in medicine and surgery now proceeding at the University.—A church parade on the part of the various friendly societies of Taunton on behalf of the District Nursing Association has realised a sum of over £51.—A three days' bazaar was held lately in Kensington Town Hall in aid of a fund for sending sick children to the seaside for a fortnight. The Duchess of Devonshire opened the proceedings on the first day, and the Countess Cadogan on the second.—"The Woman's Signal" for June 18th contains an illustrated interview with Miss Ellen Orme, the popular matron of the London Temperance Hospital.—Something seems to be seriously wrong at the Clapham Infirmary. At a recent meeting of the Board of Guardians it was stated that the assistant nurses "were underpaid, badly housed, and overworked." There have been forty resignations in fourteen months.—Eight nurses have lately resigned at the City of London Union Infirmary (Bow). It was stated at the Board meeting that the pay was as bad as the work was hard.—The troubles at the West Bromwich Workhouse have been settled by the Board finally advertising for a hospital-trained head nurse. They have taken more than three months to see the wisdom and necessity for following the instructions of the Local Government Board on this point.

### Hygiene: For Nurses.

By JOHN GLAISTER, M.D., F.F.P.S.G., D.P.H.Camb., Professor of Forensic Medicine and Public Health, St. Mungo's College, Glasgow, &c.

### XIV. — WATER SUPPLY (*continued*). — MODES OF SUPPLY. — CHARACTERISTICS OF WHOLESOME WATER.

THE present-day water supplies of the world are obtained either from (1) rivers, (2) lakes, (3) springs, (4) wells, or (5) directly from the rainfall. These demand some detailed consideration.

RIVERS.—In its virgin condition a river is but a moving column of rain-water, having in solution what the water may have dissolved in its passage through the air on its way to the river in the various rivulets, or in the river-bed itself. In this state, the water of a river is usually a good water. Hence rivers are tapped as water supplies, less, however, now than formerly. London, to some extent, is still supplied from the Thames and its tributaries. At the same time, by reason of the risks of pollution, rivers are suspici us sources of supply; as a rule, they are positively dangerous. Before the best of them can be used efficient filtration must precede distribution. It were better, indeed, if they could be done without.

LAKES are natural upland collections of water, and constitute the source of our purest supplies. Occurring in sparsely inhabited areas, they are little liable to contamination; formed on rock strata, difficult of solution, they contain but little mineral matter, and, constituted only of water from the clouds, they are "soft," and, therefore, excellent for washing purposes. The chief objection against them is that they are rich in vegetable organic matter, which is sometimes solvent of the lead of the pipes which contain and convey the water.

EXPLANATION OF FIG. 28 (From Fessenden's "Physics.")—Figure represents a vertical section of geological strata of basin. *a*, loose sand or gravel layer; *b*, clay bed; *c*, stratum of slate; *d*, stratum of limestone; *e*, a bed of granite. Upright lines show spouting bore-tubes.

RESERVOIRS are but artificial lakes, formed in similar districts and for similar reasons. They form the focussing point of the water which falls over a given portion of land, called the *gathering ground*.

SPRINGS usually supply but limited amounts of water, enough, however, in many cases, for small collections of houses.

WELLS constitute the chief source of water supply of rural districts, and of isolated houses, including farms. The purity of the water, in relation to milk supplies, becomes of prime importance. There are three kinds of wells, viz. (1) shallow or surface, (2) deep, and (3) artesian; and their designation does not so much depend on their actual depth as upon their relation to the earth-strata through which they are driven. A shallow or surface well is one which, sunk into pervious soil, taps the underground water which has percolated from the surface of the ground within the immediate vicinity of the well-shaft. A deep well is one which, sunk through the pervious surface-layer of soil and an underlying impervious stratum of rock, taps the underground water which has percolated from the land surface at some distance from the well-shaft. An artesian well (so called from Artois, a province in France, where they were first used on a large scale) is one in which the shaft is sunk through (1) a pervious layer, (2) an impervious layer, which taps a collection of water lying on the top of (3) an impervious layer, the two latter of which "outcrop" on the surface of the land, it may be, several miles away. The water which is tapped, falling on the area of land between the two outcropping layers, sinks into this basin-like formation, and lies there under considerable pressure. When the bore-tube, in the process of sinking, reaches this collection of water, the pressure of the mass of water lying between the two impervious layers forces the water up the tube and into the air occasionally. Fig. 28 illustrates the conditions requisite for this kind of well.

Artesian wells are usually of considerable depth; that of Grenelle, in Paris, is 1,800 feet; of Passy, 1,900 feet; and of Rochefort, 2,765 feet. Their waters are high in temperature, depending on the depth; that of Grenelle is 82 deg. Fahr., and that of Rochefort 106 deg. Fahr. They provide large quantities of water; that of Grenelle 500,000 gallons per 24 hours. The water from the well of Grenelle drains a district 100 miles from Paris. They supply waters free from organic pollution, but frequently containing much mineral matter in solution.

In respect of purity, waters from springs and deep wells are the best, while surface water and stored rain water are suspicious sources, and rivers and shallow wells are dangerous sources.

Rain water is sometimes collected for potable uses, but only rarely; whereas for washing purposes it is eagerly stored in districts where the other supplies are "hard," because of its "softness." We have computed the total amount of rainfall in the British Islands in an average year to be 232,000,000,000 tons of water, or 52,000,000,000,000 gallons, but a very large proportion of this finds its way back to the sea by the rivers, and to the atmosphere by surface evaporation.

HARDNESS AND SOFTNESS.—These are terms employed to express the difficulty or ease with which water unites with soap to form a lather, or, the soap-destroying power of a water. Economically, this is a matter of importance in a manufacturing community, and for domestic purposes. As a hard water requires much more soap than a soft one the increased expenditure in soap alone forms a formidable money bill in London, or in any other community supplied with a hard water. Waters differ much as to hardness or softness; for example, the water of Glasgow has about 1 of hardness, those of London from 12 to 21, and that of Sunderland 30°. This difference depends on the source of supply. Rain water is the softest, and from the following sources in progressive order they become harder, viz.: Lakes, rivers, deep wells, artesian wells. Hardness is due to the presence of salts of lime and magnesia dissolved in the water from strata containing these substances, through the agency of $CO_2$ which the rain takes up from the atmosphere in falling. The other principal factor of hardness is iron, which is dissolved in the water in the same way. Lime and magnesia, as carbonates or sulphates, or both, and iron are, therefore, the chief causes of hardness in any water. Anyone can determine between a hard and soft water by the simple act of personal ablution, but the rationale of it is not so well understood. The following facts will explain: Hard soap is a compound of a fatty substance and soda. The fatty substance is composed of fatty acids and glycerine—in union. These fatty acids unite with the alkali—soda—to form hard soap—chiefly the oleate and stearate of soda, which are soluble in water. When soap is dissolved in a water which contains lime or magnesia, or both, the lime splits up the soap and unites with the oleic and stearic acids to form these salts of lime, which are insoluble in water. Hence, so long as lime is present ununited with these fatty acids, no lather will form. For this reason, therefore, a hard water uses up more soap than a soft one. We distinguish between kinds of hardness, based solely upon the chemical nature of the lime and magnesian salts present in the water. There is "total," "temporary," and "permanent" hardness. Total hardness means the natural soap-destroying powers of a water; permanent, the hardness which remains after the water has been boiled; and temporary, that which disappears on boiling. Temporary hardness is due to the carbonates of lime, magnesia, and iron; permanent, to the sulphates of these metals. Hard waters usually contain both forms. The carbonates are kept in solution by excess of $CO_2$ in the water. By boiling, this gas is driven off, and these salts, no longer able to exist in solution, fall as a white powder, which adheres to the sides of the vessel, and accumulates to form a crust. This crust prevents a kettle or a boiler from being easily heated, therefore more coal has to be used to make the water boil or to generate steam, hence increased expenditure of money. Besides, the crust not allowing the heat of the fire to pass to the water, the iron gets very hot, and this is a common cause of boiler explosions; hence a danger to life. In "limey" districts the knowing housewife prevents the encrusting of her kettle by placing in it a large playing marble, around which the crust forms. Thus, after boiling, water containing lime, &c., as carbonates, is rendered softer. This is temporary or removable hardness. Lime and magnesian sulphates are unaffected by boiling, hence the hardness remains persistent, and is therefore called permanent hardness. Total hardness is the sum of both. To remove the hardness due to sulphates the washerwoman adds carbonate of soda, which, reacting on the lime and magnesian salts, forms sulphate of soda and carbonate of lime, which latter becomes precipitated in boiling.

So much discomfort and increased expense arise from hardness in water, that methods have been invented to overcome it, and with some degree of success. But for necessity, therefore, no populous place would choose a hard water if a wholesome soft water could be obtained.

### Lady Dufferin's Fund.

A garden fête in aid of Lady Dufferin's Fund for Supplying Medical Aid to the Women of India was held, on Thursday, July 2nd, in the grounds of Kidbrook Lodge, Blackheath. Lady Dufferin herself opened the fête, and was accompanied on the platform by Sir Alexander Wilson, Lord Lansdowne, Lady Vincent, and Lady Peile. Sir Alexander Wilson introduced Lady Dufferin, and, speaking of the origin and development of the scheme, emphasised strongly the value of its work, and the urgent necessity for cordial and substantial support being given to the United Kingdom branch, which supplied the sinews of war, funds, doctors, &c., to the main association. Lady Dufferin gave a brief account of the work accomplished by the scheme in India. All races and creeds in India were, she said, in sympathy with it. It was a national association for supplying female medical aid to the women of India, and its system of doctors, compounders, hospitals, and midwives was intended to spread throughout the empire. The Buddhist priests told their people that all who wished to enter Nirvana must subscribe to the fund, and the Bishop of Rangoon exhorted all his clergy to help the movement by every means in their power. The fête, which was organised and arranged by Mrs. Hart, who devoted to it much time and energy, was well attended, and we hope the results will be eminently satisfactory from a financial point of view.

### Grand Fete at the Middlesex Hospital.

Seldom has a more charming and picturesque scene been witnessed in the heart of London than that represented by the Middlesex Hospital last week. The occasion was a reception by the Duke and Duchess of York, when purses in aid of the new convalescent home were presented. The ceremony, at which Miss Thorold, the matron, presented the Duchess with a bouquet, passed off most successfully, and the Royal visitors expressed themselves delighted with all they saw, especially with the Princess May Ward, charmingly decorated with flowers for the occasion by Mr. William Hooper. The hospital was most fortunate in securing the assistance of some of the best firms in London and the provinces, and consequently the unusual excellence of all the decorations and arrangements. Messrs. Paul and Sons' beautiful roses were seen everywhere; Messrs. Gillow, Shoolbred, Maple, and many others supplied suitable draperies and furniture; Messrs. Brock arranged the illuminations which made the quadrangle a veritable fairyland in the evening; and Messrs. Buszard supplied the excellent refreshments. There were exhibitions of the X rays and other objects of scientific interest, and a most charming collection of valuable pictures lent by friends of the hospital. In the early evening Mr. Wilhelm Ganz conducted a concert, at which well-known artistes lent their services; and later on the band of the Royal Engineers performed amidst the fairy lights in the quadrangle. One of the most interesting exhibits of the fête was a model ward, under the charge of one of the sisters, assisted by a staff of nurses. All kinds of ingenious appliances and furniture were lent by Messrs. Heal and Son and Messrs. Maw and Son. A little band of children represented patients, thus lending an air of reality without making a spectacle of sickness. It is difficult to imagine what more could have been added to make the occasion of interest. The general effect was all that is charming, and the whole arrangements admirable, and much appreciated by the guests who thronged the building during the afternoon and evening. We are glad to learn that the presentation of purses produced a handsome sum towards the funds of the convalescent home.

### The Actors' Orphanage Fund.

Some time ago Mrs. Clement Scott and Mrs. Charles Carson formulated a plan to raise an orphanage fund in connection with the theatrical profession. Last week a bazaar in aid of the scheme was held in the Queen's Hall, Langham Place. The stalls, which were tastefully decorated, were presided over by leading actresses of the day. There were also numerous and varied entertainments, at which nearly every artiste of note in London at the time assisted. In spite of the unusually interesting programme of proceedings, the bazaar was not so well supported by the public as it might have been. There was an amusing hat trimming exhibition presided over by Mr. W. Denny, and assisted by well-known gentlemen of the stage, and Miss Lila Clay's Ladies' Orchestra played charmingly each day the bazaar was open. A beautiful Mount St. Bernard dog was present in the entrance hall, and pleaded for the orphans in a most effectual manner, and numerous contributions found their way into the green satin satchel which hung round his neck. Nothing could have exceeded the spirited endeavours of the stall-holders and their assistants to secure a success, and we could wish that the result of the sales had been larger

### Reception of "Queen's Nurses" by Her Majesty at Windsor.

THURSDAY, July 2nd, 1896, was a day which will be for ever memorable in the annals of Queen Victoria's Jubilee Institute for Nurses, when the Queen received the Council of the Institute at Windsor, together with 398 of the nurses whose names are inscribed on the roll as "Queen's Nurses." Readers of THE HOSPITAL will hardly need to be reminded of the origin of the Institute, established and endowed by the Queen with the sum of £70,000, part of the "Women's Offering" to her in the year of Her Majesty's Jubilee, with the object of bringing trained and skilled nursing into the homes of the poor in times of illness. The Institute, the head-quarters of which are at St. Katharine's Hospital, itself an ancient foundation, in Regent's Park, now numbers on its active list 539 nurses, having branches in Scotland, Ireland, and Wales, as well as throughout the rural districts of England.

By twelve o'clock on Thursday Paddington Station was thronged with blue cloaked and bonnetted nurses, whose bright, happy, and singularly healthy faces were a pleasure to look upon. They were women enjoying play because they also enjoy work. It would have been difficult to find one word of adverse criticism on the score of the appearance of Her Majesty's nurses, who were one and all exceptionally neat and nurse-like, the highest praise which can be given, and a delightful contrast to the untidy heads, flowing veils, and unkempt look of some nurses still to be seen abroad. The special train left the station at ten minutes to one, arriving at Windsor at twenty-five minutes past the hour. There, under the direction of the Hon. Sydney Holland, in whose able hands the organisation of the day's proceedings had been placed, the nurses formed in fours and marched along the terrace round the Queen's private garden (which was thrown open to them during the afternoon) to a marquee on the tennis lawn in front of the Castle, where an excellent luncheon was ready spread for them. This, by the way, was by special command of the Queen, who, hearing that the Council intended giving the nurses luncheon at the Town Hall previous to their reception by Her Majesty at five o'clock, expressed a wish to provide the whole of the day's entertainment herself. The members of the Council, meanwhile, were received at the Castle, where they lunched with the Royal Household, who spared no pains to show them every possible courtesy and kindness.

Mr. Holland, who was simply indefatigable throughout the day, and won the hearts of all the nurses by his genial fun and kind consideration for everyone's comfort, made the nurses an amusing little speech, detailing the afternoon's programme, and practised them (by way of "grace") in the first verse of the National Anthem, which they were to sing by-and-by. Luncheon over, parties of fifty were taken through the State apartments, and by special permission through a part of the library, returning at a quarter to five to the tennis courts. The weather was a little threatening, but fortunately no rain actually fell, so it was possible to abandon cloaks, and the scattered crowd of dark blue gingham dresses, with spotless white aprons, and neat, dark blue, veilless bonnets and white strings, made a pretty picture, with the grand old castle, of which all English people are so justly proud, in the background. The superintendents wore dark blue alpaca dresses, without aprons, and all were distinguished by the armlet with its V.R.I. and the badge on its blue and white cord or ribbon round their necks. It was a little break in the harmony that some of the nurses from affiliated associations do not wear the real "Queen's" uniform, and so there were lilac print dresses from East London and strawberry-coloured ones from Westminster, while the Manchester nurses wore green cloaks and bonnets instead of the regulation blue. There was a Scotch contingent, sixty-nine in number, twenty nurses from Ireland, and quite a large group represented Wales.

Then came a dress rehearsal. The nurses were drawn up in three sides of a hollow square, facing the Kennels Road, the Scotch, Irish, and Welsh nurses on the right wing, the English forming the centre and the left wing. "I'm Monmouth; am I Wales or England?" asked one bright-faced nurse, with much enjoyment of the joke, and she was sent to join the Welsh group in spite of a protest. The nurses were quickly in order, answering with a prompt and intelligent obedience to the word of command, which testified to the value of a trained and disciplined habit of mind. Drilled by Lord Alwyne Compton and Mr. Holland (who might have been seen demonstrating "curtsies" in the midst of laughing groups of nurses earlier in the day), the approach of the Queen in a carriage drawn by grey horses was greeted by the nurses withlow, simultaneous curtsies, very striking in effect. Her Majesty was accompanied by Princess Christian of Schleswig-Holstein and her daughter, Princess Victoria, being joined on the ground by the Princess Louise (Marchioness of Lorne), the Marquis of Lorne, K.T., and Prince Alexander and Princess Victoria Eugénie of Battenberg. The carriage stopped facing the centre of the square, and Sir John MacNeill, one of the Queen's equerries, introduced to Her Majesty the Master of St. Katharine's Hospital, the Rev. Arthur L. B. Peile, who is by virtue of that office president of the Jubilee Institute. The Master then presented Mr. Rathbone (vice-president), Sir James Paget (one of the three trustees of the Institute), and the following members of the Council: Lady Lucy Hicks-Beach, Lady Victoria Lambton, Lady Penrhyn, Mrs. Grenfell, Mrs. Theodore Acland, Mrs. Power-Lalor, Miss Rosalind Paget (late hon. inspector), the Earl of Meath, Lord Balcarres, Lord Alwyne Compton, the Hon. Sydney Holland, Sir Dyce Duckworth, Mr. Bonham Carter, Mr. Samuel Hoare, M.P., Mr. Thomas Bryant, and the Rev. Dacre Craven. Miss Guthrie Wright, hon. secretary of the Scottish branch, was presented to the Queen by Princess Louise, its president, and the inspector (Miss Peter), the assistant inspector (Miss Sara Peter), Miss Dunn (superintendent for Ireland), Miss Wade (superintendent for Scotland), and Miss Oldham (for the rural district branch), by the Master. The presentations completed, the nurses sang "God Save the Queen" with a hearty spontaneity which Her Majesty must have appreciated.

It is very rare for the Queen to change arrangements upon which the seal of her approval has once been set, but on this occasion the programme was specially altered by Her Majesty in order that she might personally speak to her nurses, a mark of very particular interest and honour. Driving into the very centre of the square the carriage drew up, and the Queen, raising her voice, said clearly and distinctly, "I am very pleased to see my nurses here to-day, and to hear of the good work which they are doing, and which I am sure they will continue to do." Those who were near enough to see Her Majesty's face felt very certain that the pleasure she afterwards expressed to the president at the sight of her nurses and their general appearance, was deep and real. The whole ceremony, indeed, was one never to be forgotten by those who witnessed it, and not the least impressive sight was the final march past of the nurses, by the Queen's particular desire. Then returning to their places they curtsied once more as the Royal carriage drove away down the road towards the Kennels.

Tea was by this time ready in the tent, with a lavish and much-appreciated supply of fruit and cream, and other good things, and then came the fun of being photographed. Many a group was taken which will be treasured for years to come, and exhibited to admiring patients and friends as mementos of "our splendid day at Windsor." Leaves and daisies, too, were plucked, to be carefully put away as sacred relics by their happy possessors, and then it was time to be once more on the march, this time up the steps to the north terrace and away down to the station, four abreast, stopping *en route* at St. George's Chapel, from which the last batch of nurses was extracted with difficulty in time to reach the station by the appointed time. The inhabitants of Windsor turned out to watch with interest the long stream of nurses coming down the steep hill from the Castle. It was a train full of happy, if rather tired, women who reached Paddington a little before eight, to be conveyed thence to Grosvenor House, where, at the invitation of the Duke and Duchess of Westminster, they were to spend the rest of the evening.

Much amusement was caused by the sight of Mr. Holland laden with "lost property," caps and stray cuffs, umbrellas and cloaks, driving after the procession of omnibuses arriving in time to restore their possessions to the rightful owners, and to help the nurses from their exalted positions on the roofs, with the aid of several stalwart policemen, who seemed to enjoy their unwonted duties. "Being angels," said someone, "they know not how to descend!"

At Grosvenor House.

A musical treat was in store for the nurses at Grosvenor House. The Artillery band, stationed in an alcove at the end of one of the large reception rooms, played charmingly; and presently, after the arrival of the members of the Council and other guests, Mons. Johannes Wolff appeared, and made his violin speak lovely things. Madame Albani was hailed with enthusiasm, and her singing of "Home, Sweet Home" perhaps gave the keenest pleasure of all to the nurses. The programme was most enjoyably completed by several songs from Mr. Plunket Green.

Her Royal Highness Princess Louise, who took the most cordial and personal interest in everything, and had hurried back from Windsor in order to be present, arrived early, speedily followed by the Duke and Duchess of Teck.

The music over, the Master of St. Katharine's made a brief speech. The Queen's nurses, he said, needed no incentive to do their duty, but such a display of gracious favour as that extended to them by the Queen that day would send them back to their various districts more than ever resolved to do their best for the help of the sick and suffering poor. He repeated once more Her Majesty's words to her nurses, and commented upon the striking fact that in that gathering there were nurses from all parts of the United Kingdom, and that most of them had been especially charged by their poor patients with messages to the Queen of loyalty and affection, showing how grateful they were for the kindly thought and care which provided for them a body of competent, trained women to minister to their needs. He hoped to-morrow the account of the day's proceedings would go forth to the public, and the world would know that there were no women who deserved more cordial recognition than these nurses. The presence of their Royal Highnesses there that night was a real encouragement to them, and the nurses would go back to their work feeling that it was indeed appreciated. He proposed a vote of thanks to the Duke and Duchess of Westminster, their kind host and hostess, and to Mr. Holland, who had contributed so much to the successful carrying out of the day's arrangements. (Applause.) In reply the Duke of Westminster in a few words expressed the very great pleasure it had given him to be able to contribute to their enjoyment. Then the nurses were marshalled to supper, the Duke and Duchess of Westminster personally seeing to their comfort and enjoyment. The day was an unqualified success, and will be treasured in the remembrance of all the nurses who had the good fortune to be present.

Any inquiries about property lost or fares over-charged, &c., should be made to the Secretary of the Queen's Jubilee Institute, Miss Leake, St. Katharine's Royal Hospital, Regent's Park. Miss Leake will also be glad to communicate with the photographers for any nurses who did not order photographs when at Windsor, but wish to do so.

LETTER FROM THE PRESIDENT.

We have received the following letter from the Master of St. Katharine's (the Rev. Arthur L. B. Peile), president of the Queen's Institute:—

"Will you allow me through your columns to thank, in the name of the Council of the Queen's Institute, the committees of the associations affiliated to the Institute (nearly 300) for the ready permission and generous assistance which was given to enable the Queen's nurses who are employed by them, to assemble at Windsor Castle last Thursday? It was a day which will not be forgotten by those who were present, and we are greatly indebted to the various local committees in all parts of the United Kingdom for enabling the council to present before the Queen nearly 400 nurses who are on the roll of the Institute. A few were, to their very great disappointment, at the last moment prevented from attending, having cases of sickness on hand which could not be safely left. The Council thank these nurses for their self-denying devotion to the sick poor. The necessity for careful nursing in sickness the suffering poor in their own homes cannot fail to claim the attention which it deserves, seeing that Her Majesty has given such public expression of her approval of the work which her nurses 'are doing and will continue to do.'"

### New Queen's Nurses.

Her Majesty the Queen has been pleased to approve of the following names being placed on the "Roll of Queen's Nurses":—

England.—Superintendents: Charlotte Tenney, serving at Woolwich; Wilhelmina Dow, Portsmouth. Nurses: Jessie Coleman, serving at Camberwell, S.W.; Agnes James, Droylsden; Harriet A. Crees, East London; Emma Pickersgill, East London; Elizabeth C. Aylward, East London; Amy H. Barchard, East London; Ada M. Bevan, Rochdale; Birdsall Hobkinson, South London; Alice Bridgford, Kensington, S.W.; Emily J. Tipping, Rotherhithe, S.E.; Elsie H. Holloway, Paddington, W.; Florence Steele, Haggerston, N.E.; Annie S. Dunne, Haggerston, N.E.; Amy B. Hill, Haggerston, N.E.; Sarah A. Andrews, Rawtenstall; Alice A. Brown, Southampton; Agnes Tyson, Hull; Ellen E. Gerring, Leeds; Mary A. Gravert, Hebden Bridge; Florence L. Butler, Malvern; Hilda Goadby, Tunbridge Wells; Eleanor Purton, Bath; Sarah E. Hutchinson, Bath; Caroline M. S. Reid, Windsor; Annie Owen, Trevor; Beatrice Drayton, Leamington; Mabel L. Orgias, Rochdale; Annie Handley, Warwick; Agnes MacCallum, Clutton; Clara Urridge, Bognor; Ann M. Clough, Manchester; Florence J. Harrison, Manchester; Emily Ridsdale, Manchester; Elsie M. Boss, Manchester; Elizabeth Evans, Manchester; Elizabeth J. S. Hazelton, Manchester; Florence Grundy, Manchester; Agnes L. Ross, Salford; Emily Traiforos, Salford; Mary Bull, Salford; Blanche E. Hancock, Cardiff; Amelia Johnson, Haverfordwest; Alice E. Pennington, Bangor.

Scotland.—Nurses: Mary McGuffie, Edinburgh; Lucy J. Carey, Edinburgh; May Lindsay, Glasgow; Hughetto E. Tennent, Glasgow; Mary A. Hill, Glasgow; Janet Carmichael, Glasgow; Jessie Cran, Aberdeen; Helen Largue, Paisley; Mabyn Armour, Paisley; Mary M. Easton, Arbroath; Jane C. Robertson, Motherwell; Margaret A. Fyffe, Motherwell; Isabel Jack, Ballantrae; Mary Murray, Musselburgh; Bertha Lawton, Slateford; Margaret J. Nesbit, Ratho; Helen Beechie, Collinton; Rachel Taylor, Alyth; Adelaide Thackeray, Port Glasgow; Mary Kelly, Lossiemouth.

Ireland.—Nurses: Annie Fleming, Dublin; Florence R. Gardiner, Londonderry; Jane McCotter, Cushendall; Mary O. Fitzsimon, Newbridge; Annie Walsh, Coole, Mayne, and Kiltoom.

### The St. Olave's Guardians and the Matron of the Infirmary.

IT is no new fact to our readers that the conduct of nursing matters in union infirmaries is far from being universally satisfactory. At the St. Olave's Infirmary the nursing department has not worked well for some time, and the unsatisfactory state of affairs has ended in the resignation of Miss Evans, the matron. In justice to Miss Evans and in the public interest we publish an account of the events preceding this resignation. Miss Evans informs us that for some time past, finding that the conduct of the nursing at St. Olave's was becoming more difficult, and for private reasons of her own, she decided to resign her appointment. But before she could send in her resignation she received a letter from the Guardians themselves asking her to resign, and enclosing her a copy of the resolution passed by the board to the following effect:—

"That, having regard to the past history of the matron of the Infirmary, the general incapacity she has shown in the performance of her duties, her disobeyance of the orders of the Guardians, and her want of co-operation with the Visiting Committee in the administration of the Infirmary, Miss M. M. Evans be requested to forthwith submit her resignation as matron of the Infirmary."

On receiving this letter Miss Evans felt it would be against her interests to comply with the desire of the Guardians, for she thought an inquiry into the charges made was due to herself. She therefore declined to resign. Now, a Board of Guardians has no power to dismiss a matron from their infirmary, but they may suspend her, and this the St. Olave's Guardians proceeded to do, and reported the suspension to the Local Government Board. The Local Government Board informed Miss Evans that they had received an intimation from the Guardians, and enclosed her a copy of the general charges made against her. We think that it is due to Miss Evans to publish the reply which she sent to the Local Government Board meeting these charges, which was as follows:—

St. Olave's Infirmary, 16th April, 1896.

Sir,—I have the honour to acknowledge the receipt of your letter of 10th April, 1896. The resolution of the Guardians of St. Olave's Union (12th March, 1896) calling on me to resign the office of matron which I have held for six years embraces general charges against me under four heads, viz.: (1) Past history; (2) general incapacity; (3) disobedience of orders; (4) Want of co-operation with the Visiting Committee in the administration of the infirmary.

1. With reference to my past history, I am quite unaware that there is anything in it which will justify any censure from the Guardians, or on account of which I ought to resign my appointment.

2. As to the charge of general incapacity, I think I may refer to my official record at St. Olave's Infirmary, at Stockport Infirmary, at the Northern Hospital, Liverpool, and at the London Hospital. I must not offer any opinion as to my own capacity, but I refer without hesitation to the medical superintendent of St. Olave's Infirmary (Dr. A. R. Johnston), to the committee of the Stockport Infirmary, and to those of the inspectors of the Local Government Board who have been acquainted with my work.

3. As to disobedience of the orders of the Guardians I am not aware of any case in which I have failed to carry out the orders of the Guardians except on two occasions, viz., once when the Guardians sought to restrict my private correspondence, a matter in which I conceived they had no concern; and once again when they directed me to sign a paper acknowledging that in the administration of the infirmary I was subservient to the steward. On this point I understand that under the system of the Local Government Board for the administration of infirmaries, amongst the officers the matron is subordinate only to the medical superintendent, and has precedence over, or, at the lowest, co-ordinate rank with, the steward.

4. It has been my constant effort to work in co-operation with the Visiting Committee, and to assist them to the utmost of my power in the supervision of the infirmary. I am not conscious of having failed in this duty. But there have been some things connected with individual members of the Visiting Committee which appeared to me to be in no sense supervision, and to which I felt it my duty to object. For instance, it has appeared to me undesirable, and not conducive to efficiency or discipline, that individual members of the committee should take tea in a nurse's duty-room, and keep her from her patients; or that members of the committee should be smoking in the nurse's sitting-room at midnight; or that young nurses and probationers should go to smoking-concerts (even though Guardians and officers of the infirmary were present), and remain out until after midnight; or that nurses should have a general pass to remain out until half-past eleven p.m. To these matters I have felt it my duty to object. The Guardians in their resolution have not given any details of their complaints against me, but I am fully prepared to answer any detailed or particular charges should the Local Government Board think it well to call for them. I need only add that it is, and has always been, my anxious desire to carry out my duties thoroughly, conscientiously, and efficiently, and in entire harmony with the Guardians of the union, and with all the officers of the infirmary.—Your obedient servant,

M. M. EVANS.

This letter, or part of it, the Guardians received from the Local Government Board, with a request that they should make their observations upon it. In the meantime, Miss Evans' own private affairs imperatively demanded her attention, and the long delay which followed rendered Miss Evans' position both trying and inconvenient. So, feeling that a full inquiry into the charges was being made, she resigned her post.

On June 4th the Guardians sent a long statement of charges against Miss Evans to the Local Government Board. We are unable to give any details as to this statement, as it was not sent on to Miss Evans by the Local Goverment Board, who wrote to the Guardians the following letter, of which they sent a copy to Miss Evans:—

Local Government Board, Whitehall, S.W.,
30th June, 1896.

SIR,—I am directed by the Local Government Board to acknowledge the receipt of your letter of the 4th instant forwarding a copy of a letter addressed to the Guardians of the St. Olave's Union by Miss Evans resigning the office of Matron of the Infirmary. As Miss Evans has resigned, the Board do not propose to deal in this letter at any length with the charges brought against her, but they cannot allow the matter to end without stating that they in no way agree with the course adopted by the Guardians. The Board have carefully considered your letter of the 27th ultimo, and the printed extracts from the minutes which accompanied it, but they do not find adequate foundation for the charges brought against Miss Evans. It appears to the Board that the wording both of the letter and of the marginal notes of the printed minutes is, in more than one instance, unwarranted by the facts and unfair to Miss Evans. On the other hand, the Board are unable to avoid the conclusion that the Guardians have evinced a want of due consideration for the Board's position, and this must, in the Board's view have had a detrimental effect on the discipline and administration of the infirmary. The Board would also advert to the following statement on page 26 of your letter: "As to the reference to young nurses and probationers going to smoking concerts (even though Guardians and officers of the infirmary were present), and remaining out till after midnight, the Guardians submit that it is not their place, or the duty of a matron, to control their officers and servants when they are off duty, or to make any inquiries as to their whereabouts." The Board desire me to express their strong dissent from the views thus stated, and they trust that, upon further consideration, the Guardians will realise that the conduct and well-being of their officers at all times is a matter which should deeply concern them. . . . .—I am, &c.,

(Signed) W. E. KNOLLYS, Assistant Secretary.

Miss Evans cannot possibly wish for a completer vindication of her actions than that which the letter from the Local Government Board affords. It exonerates her from the blame which the Guardians attach to her name, and leaves untouched the matters which she brought against them. We can only hope that Miss Evans' successor, and the patients and nurses under her charge, will reap the benefit of the exceedingly unpleasant experiences through which she has passed.

### Novelties for Nurses.

NURSES' REQUISITES.

We are often asked by nurses where various appliances and requisites appertaining to their calling can be obtained. We are always very pleased to comply with these requests, but feel we shall be benefiting a much larger number by calling attention to such useful catalogues as that of which Messrs. Maw, Son, and Thompson have sent us a copy. The "Nurses' Handbook," as it is calle1, is fully illustrated and indexed. Within it will be found, not only the useful list of nursing requisites, but also a short dictionary of medical terms, and recipes for invalid cookery, besides much other information relating to nursing. Amongst the sick-room necessaries we note some excellent models of inhalers, steam kettles, and sick-room furniture. For nurses, the selection of dressing instruments and convenient wallets is very complete. The useful work can be obtained from Messrs. Maw, Son, and Thompson, 7, Aldersgate Street, E.C.

### Appointments.

Herne Bay Convalescent Home (South Metropolitan School District).—Miss Emily Turton has been appointed Matron at this institution. She received her training at Guy's Hospital and at the Leeds Trained Nurses' Institute. Miss Turton worked as nurse in connection with the Leeds Institute, and afterwaids held the post of night sister and that of sister-in-charge at the Grimsby General Hospital.

Basford Isolation Hospital, near Nottingham.—Miss E. M. Pringle has been appointe1 matron of this hospital. She was trained at the Adelaide Hospital, Dublin, afterwards working as a private nurse in connection with the same institution. Miss Pringle held the position of sister at Stillorgan Convalescent Home, Dublin, for a year, and has since worked as charge nurse in the Eastern Fever Hospital, Homerton, London. We cordially wish her all success.

### Notes and Queries.

### Queries.

*Royal National Pension Fund for Nurses.*—Nurse Alice is reminded that queries must be accompanied by name and address.

(100) *Military Nursing.*—Please tell me how I can get into Netley Hospital, and to whom I should apply?—*Nurse B.*

(101) *Infirmary Training.*—If I train for four years at a workhouse infirmary, shall I be recognised as a fully-trained nurse, and be able to obtain work in a good general hospital afterwards?—*Sunnyside.*

(102) *Holidays.*—Can you tell me of any nurse midwife who would be willing to undertake district work for three or four weeks for a small salary in Cheshire?—*Nurse J.*

(103) *Home Wanted.*—Could you tell me of a home where a girl of 17, suffering from spinal disease, could be received for a small payment?—*Liverpool.*

(104) *District Nurses.*—Can you tell me where I am likely to hear of well-trained district nurses, trained specially for that branch of the work?—*Superintendent.*

(105) *Holidays.*—Can you tell me of any reasonable lodgings in Cromer or within a walking distance of that place?—*M. A. L.*

### Answers.

(100) *Military Nursing* (*Nurse B.*).—Write to the Director General of the Army Medical Department, Victoria Street, Westminster, S.W. Three years' training in a good general hospital is a necessary qualification for an army nursing sister.

(101) *Infirmary Training.*—Certainly training in a good poor law infirmary under a matron who is herself a trained nurse is recognised. Large general hospitals train their own nurses, and have, as a rule, no need to take outsiders. In smaller institutions, where there may be occasional vacancies on the staff, it might be possible to obtain an appointment.

(102) *Holidays* (*Nurse J.*).—Watch the advertisements in The Hospital "Nursing Supplement," and write to the secretary of the Midwives' Institute and Trained Nurses' Club, Mrs. Nichol, 12, Buckingham Street, Strand, W.C., enclosing stamped envelope for reply. She may possibly be able to help you.

(103) *Home Wanted* (*Liverpool*).—You will find a list of suitable homes in Burdett's "Hospitals and Charities," Scientific Press, 428, Strand, W.C.

(104) *District Nurses* (*Superintendent*).—Have you advertised? We do not know how else you would be likely to hear of the right people. How about affiliation with the Queen Victoria Jubilee Institute?

(105) *Holidays* (*M. A. L.*).—Write to the Great Eastern Railway (2, Cockspur Street, S.W.) for their small pamphlet, "Holiday Notes in East Anglia." It gives most useful information, and will help you about lodgings.

### For Reading to the Sick.

SYMPATHY.

**Motto.**

The truest joys which we have experienced have come when we have had grace to enter most entirely into a sorrow not our own.—*Wescott.*

**Verses.**

Speak kindly to thy fellow-man,
Lest he should die, while yet
Thy bitter accents wring his heart,
And make his pale cheek wet.
Speak to him tenderly, for he
Hath many toils to bear;
And he is weak, and often sighs—
As thou dost—under care.
Speak to him lovingly, he is
A brother of thine own;
He well may claim thy sympathies,
Who's bone of thine own bone.

—*From "Heavenly Thoughts."*

There is no sorrow, Lord, too light
To bring in prayer to Thee;
There is no anxious care too slight
To wake Thy sympathy.
Man who has trod the thorny road
Wilt share each small distress;
The love which bore the greater load
Will not refuse the less.
There is no secret sigh we breathe
But meets Thine ear Divine;
And every cross grows light beneath
The shadow, Lord, of thine. —*J. Crewdon.*

**Reading.**

"Men are born to be serviceable to one another; therefore, either reform the world or bear with it."—*Marcus Aurelius.*

Sympathy should especially be brought out in us by sickness. No sick persons have truly understood the lesson that it was designed to teach them, until they have learned this truth. They may deeply feel their deficiences, for "we are all by nature hard and unsympathizing." We are very slow to learn true sympathy. It is very easy to sympathize with some persons who suit our tastes, and with such trials as are exactly like our own. But this is but a form of self-love and selfishness. In sympathizing with them, we seem, as it were, to sympathize with ourselves; we never forget their relation to us; thoughts of self run through the whole. Sickness, wrongly received, increases selfishness to the highest degree. Sickness, rightly received, does, by degrees, cast out the "unclean spirit," whose "name is legion." Sympathy is not natural to us; it can only be given to us by our sympathizing High Priest, but as He was "perfected through suffering," so He perfecteth us.

The way to get increase of sympathy is to seek for increase of charity. "The essence of sympathy is charity." No one without true charity can have godly sympathy. The more we are conformed to the image of perfect love the more we really understand, and seek to practise, St. Paul's description of charity, the truer, the more abiding, and the deeper will be our sympathy.

### Minor Appointments.

St. Saviour's Infirmary, East Dulwich Grove, S.E.—Miss Florence Monkhouse has been appointed Ward Sister at this Infirmary. She was trained at St. George's Hospital, and has since held an appointment as charge nurse at the South-Eastern Hospital, New Cross.

Brentford Union New Infirmary.—Miss Bessie Kennedy has been appointed Assistant Matron at this infirmary. Miss Kennedy was trained at the St. Marylebone Infirmary, Notting Hill, and has since held appointments as nurse at the Edinburgh Royal Infirmary and at the City Hospital, Edinburgh.

## Figures and Tables

**Figure f1:**